# Comparison between the triamcinolone and bevacizumab subconjunctivals and changes in Interleukin-1 mRNA expression in pterygium

**DOI:** 10.1016/j.jtumed.2021.07.009

**Published:** 2021-08-16

**Authors:** Purnamanita Syawal, Budu Budu, Mochammad Hatta, Muhammad Nasrum Massi, Andi Muhammad Ichsan, Rahmawati Minhajat

**Affiliations:** a Department of Opthalmology, Public Eye Centre Makassar, Indonesia; b Department of Ophthalmology, Faculty of Medicine, Hasanuddin University, Makassar, Indonesia; c Department of Molecular Biology and Immunology, Faculty of Medicine, Hasanuddin University, Makassar, Indonesia; d Division of Haematology Medical Oncology, Department of Internal Medicine, Faculty of Medicine, Hasanuddin University, Makassar, Indonesia

**Keywords:** بيفاسيزوماب, انترلوكين-١ حمض ريبونيوكليك المرسال, الظفرة, الحقن تحت الملتحمة تريامسينولون., Bevacizumab, mRNA IL-1, Pterygium, Subconjunctival injection, Triamcinolone

## Abstract

**Objectives:**

Pterygium is a fibrovascular external ocular mass that grows from the conjunctiva into the cornea. The effect of subconjunctival injection of triamcinolone and bevacizumab has been inadequately investigated worldwide. This study aims to analyse the expression of IL-1 after the injection of triamcinolone and bevacizumab subconjunctiva.

**Methods:**

All patients are randomized into three groups: the triamcinolone, bevacizumab group, and placebo groups, with 5 patients in each in group. All subjects are injected subconjunctivally one week before surgery, and then surgery is performed with the autograft technique. The main outcome measures include changes in the IL-1 mRNA expression between the triamcinolone, bevacizumab, and placebo groups.

**Results:**

All samples are completed after one month of follow-up. The changes in blood levels of mRNA IL-1 expression are as follows: 4.81 ± 0.52 in the bevacizumab group, 3.40 ± 2.63 in the triamcinolone group, and 1.08 ± 1.48 in the placebo group (*p* = 0.04). In the comparison between groups, there is a significant effect between the bevacizumab and placebo groups, 3.73 ± 1.12 (*p* = 0.00), with no significant effect in the triamcinolone group, 1.40 ± 1.12 (*p* = 0.06).

**Conclusion:**

The subconjunctival injection of bevacizumab and triamcinolone before surgery is effective in suppressing inflammation in pterygium**.**

## الملخص

**أهداف البحث:**

الظفرة هي كتلة العين الخارجية الليفية الوعائية التي تنمو من الملتحمة إلى القرنية. لم يتم التحقيق في تأثير الحقن تحت الملتحمة لتريامسينولون وبيفاسيزوماب بشكل كاف في جميع أنحاء العالم. تهدف هذه الدراسة إلى تحليل تعبير انترلوكين-١ بعد حقن تريامسينولون بيفاسيزوماب تحت الملتحمة.

**طرق البحث:**

تم تقسيم جميع المرضى بصورة عشوائية إلى ثلاث مجموعات. كل ٥ عيون في مجموعة تريامسينولون ومجموعة بيفاسيزوماب ومجموعة الدواء الوهمي. تم حقن جميع الأشخاص تحت الملتحمة قبل أسبوع واحد من الجراحة، ثم أجريت الجراحة بتقنية الطعم الذاتي. تضمنت مقاييس النتائج الرئيسية تغييرات في تعبير انترلوكين-١ حمض ريبونيوكليك المرسال بين مجموعة تريامسينولون، بيفاسيزوماب، ومجموعات الدواء الوهمي.

**النتائج:**

تم الانتهاء من جميع العينات بعد شهر واحد من المتابعة. كانت التغيرات في مستويات الدم لتعبير انترلوكين-١ حمض ريبونيوكليك المرسال في مجموعة بيفاسيزوماب ٤.٨١ ± ٠.٥٢، وكانت مجموعة تريامسينولون ٣.٤٠ ± ٢.٦٣، وكانت مجموعة الدواء الوهمي ١.٠٨± ١.٤٨، على التوالي. في المقارنة بين المجموعات، كان هناك تأثير معنوي بين مجموعة بيفاسيزوماب ومجموعة الدواء الوهمي، ٣.٧٣ ± ١.١٢، مع عدم وجود تأثير معنوي في مجموعة تريامسينولون، ١.٤٠± ١.١٢.

**الاستنتاجات:**

كان الحقن تحت الملتحمة بيفاسيزوماب وتريامسينولون قبل الجراحة فعالا في قمع الالتهاب في الظفرة.

## Introduction

Pterygium is a fibrovascular conjunctiva that grows from the limbus to the corneal surface.^1^ Although the exact cause remains unclear, there is a relationship between prolonged exposure to UV light and the pterygium mechanism. Studies on molecular mechanisms, such as proliferation cell factors, inflammatory mediators and growth factors, matrix metalloproteinases, and angiogenesis, have identified factors related to the pathomechanisms of pterygium.^2^, ^3^, ^4^, ^5^ Epidemiological studies have shown that ultraviolet B (UV B) is the major risk factor for pterygium recurrence^5^^,^^6^ and triggers inflammation and progressive fibrovascular proliferation on the ocular surface after long-term exposure.^5^ The recurrence rate post excision of pterygium cases was still high (30%–90%), and 97% occurred within one-year period post-excision.^7^ Chronic UV light creates limbal basal stem cell barrier dysfunction resulting in conjunctival epithelial cell spreading to the cornea and a subsequent upregulation of proinflammatory cytokine such as Interleukin 1.^8^ The inflammation process in the conjunctiva is one of the factors involved in the recurrence process associated with pterygium. The role of corticosteroids in preventing recurrence of pterygium is well known. Triamcinolone is a medium-potency steroid that plays an important role in inflammation by decreasing fibroblast activity^9^ and inhibiting angiogenesis by vascular endothelial cells and other cells in pterygium tissues.^10^ Intraoperative subconjunctival triamcinolone injection has been shown to be effective in inhibiting the recurrence of pterygium.^11^ Another factor involved in the progression of pterygium is increasing growth factor vascular endothelial growth factor (VEGF).^12^ Bevacizumab is a humanized monoclonal antibody that inhibits VEGF-A, which stimulates angiogenesis.^11^^,^^13^ Intralesional administration has been proven to decrease pterygium size up to 14.47%.^13^ In 2017, Gupta et al.^11^ revealed that subconjunctival administration of 2.5 mg of bevacizumab was effective in preventing the recurrence of pterygium. This study aims to analyse changes in the expression of messenger RNA (mRNA) interleukin-1 (IL-1) after injection of 20 mg of triamcinolone and 2.5 mg of bevacizumab subconjunctiva in pterygium patients.

## Materials and Methods

### Study design and subjects

We consecutively enrolled 15 adult patients (30–45 years old) who had a clinically confirmed diagnosis of stage II primary pterygium according to the Bhargava et al. classification.^14^ The 15 eye patients underwent a series of examinations, such as visual acuity and slit lamp examination, to determine the type and stage of pterygium, intraocular pressure, blood pressure, and random blood sugar before injection. Exclusion criteria were infection or any ocular surface disease, systemic disease (such as diabetes mellitus, hypertension, autoimmune disorders), and previous ocular/pterygium surgery. Patients were randomized into three groups (bevacizumab, triamcinolone, and placebo groups) consisting of 5 patients for each group. Each eye received a subconjunctival injection of 2.5 mg of bevacizumab, 20 mg of triamcinolone, or a placebo (depending on the group) seven days prior to pterygium excision.

### Surgical procedures

All 15 eye pterygium patients underwent surgery by a single surgeon (P) with the autograft conjunctival technique. First, after anaesthesia with lidocaine was administered, there was a 2% injection into the subconjunctiva, followed by an injection in the pterygium area, which was approximately 5 mm from the corneal limbus. After that, we started to excise and remove the pterygium tissue from the apex to the body, leaving a triangular-shaped bare sclera. Then a conjunctival graft was made, with excision of the autolimbal conjunctiva from the superior bulbar conjunctiva. Next, a graft was used to cover the pterygium excision and was sutured using Vicryl 8–0. Post-operatively, antibiotics and steroid eye drops (Cendo Xitrol^@^) were administered four times daily. Evaluation was performed on the first day, after seven days, and four weeks after surgery to assess signs of inflammation and recurrence post-operatively.

### Sample collection and measurement of interleukin-1 mRNA expression

Blood samples were collected one week prior to surgery and one month after surgery. The samples were stored at −20 °C until assayed.

The mRNA expression of IL-1 was measured using real-time PCR according to the methods of previous studies.^15^, ^16^, ^17^

### Statistical analysis

All data are presented in the table and expressed as the mean ± standard deviation (SD). Statistical analyses were performed using Statistical Package for Social Science (SPSS) version 21.0. Changes in the mRNA expression of IL-1 using the chi square and Kruskal–Wallis tests were used. The differences between groups were tested using one-way ANOVA with a post hoc Least Significant Difference (LSD) test. A value of p < 0.05 was considered a statistically significant result.

## Results

Fifteen patients receiving the aforementioned eye treatment received follow-ups for a period of one month. The Kruskal–Wallis test for the changes in IL-1 mRNA was significant at p = 0.036. Changes in IL-1 mRNA expression with the Kruskal–Wallis test were significant at p = 0.036. The highest changes in IL-1 mRNA levels were found in the bevacizumab group. There were significant effects on blood level changes in mRNA IL-1 expression in the three groups: in the bevacizumab group, it was 4.81 ± 0.52; in the triamcinolone group, it was 3.40 ± 2.63; and in the placebo group, it was 1.08 ± 1.48 (p = 0.04) (Table 1).

**Table 1:** Level changes in IL-1 mRNA expression after subconjunctival injection of each agent.

Group	N	Mean	Std. Deviation	Std. Error	95% Confidence Interval for Mean		Minimum	Maximum
					Lower Bound	Upper Bound		
Bevacizumab	5	4.81	0.52	0.23	4.16	5.45	4.35	5.63
Triamcinolone	5	3.40	2.63	1.17	0.14	6.67	0.17	5.84
Placebo	5	1.08	1.48	0.66	−0.76	2.92	0.00	3.04
Total	15	3.10	2.28	0.59	1.83	4.36	0.00	5.84

In the comparison between groups, there was no significant effect in the triamcinolone group, 1.40 ± 1.12 (p = 0.06), whereas in the bevacizumab group and placebo group, there was a significant effect, 3.73 ± 1.12 (p = 0.00) (Table 2).

**Table 2:** Comparison of mRNA IL-1 blood level changes between groups.

(I) Group	(J) Group	Mean Difference (I-J)	Std. Error	Sig.	95% Confidence Interval	
					Lower Bound	Upper Bound
Bevacizumab	Triamcinolone	1.40	1.12	0.23	−1.03	3.83
	Placebo	3.73∗	1.12	0.00	1.30	6.16
Triamcinolone	Bevacizumab	−1.40	1.12	0.23	−3.94	1.03
	Placebo	2.33	1.12	0.06	−0.11	4.77
Placebo	Bevacizumab	3.73∗	1.12	0.00	−6.16	−1.30
	Triamcinolone	−2.33	1.12	0.06	−4.77	0.11

∗The mean difference is significant at the 0.05 level.

## Discussion

In this study, we found, as shown in Table 1, that the level of IL-1 mRNA in the bevacizumab group was higher than those of the triamcinolone and placebo groups, and there were significant changes in IL-1 mRNA expression in the bevacizumab group. In 2008, Bahar et al.^18^ used the same dose of bevacizumab as in our study and found that it did not result in long-term vascular regression in the cornea for recurrent pterygium. In 2010, Razeghinejad^19^ reported that intraoperative subconjunctival bevacizumab was not effective in pterygium recurrence. These results support our results because IL-1 is a proinflammatory cytokine that plays a role in inflammation, which is a major factor in post-operative pterygium recurrence.

Our study administered triamcinolone before surgery, whereas Kheirkhah et al. (2013^20^) administered an intraoperative triamcinolone injection and showed that it did not significantly reduce conjunctival inflammation. This result could also support our study results that triamcinolone treatment did not reduce the mRNA expression of IL-1, which led to inflammation and pterygium recurrence. Our results were also contrary to those of other studies about bevacizumab and triamcinolone. Castañeda (2015^21^) reported that a triple subconjunctival injection of 2.5 mg/0.1 mL of bevacizumab (first day, 15 days, and four weeks after the first injection), and surgery using a conjunctival autograft procedure can prevent pterygium recurrence. In our study, only one injection of bevacizumab was administered one week before surgery. Nuzzi (2017^22^) showed that bevacizumab injection subconjunctivally one week prior to surgery had a lower recurrence rate than the control. Although this result seems to contradict our result, in their study, they also reported a 7.14% recurrence after bevacizumab injection. This means that the inflammation process could still occur after bevacizumab injection and accordingly with IL-1 as a cytokine. Gupta RK (2017^11^ revealed that using triamcinolone and bevacizumab as adjuncts can result in undesirable side effects. Mpyet (2000^23^) also found that combined subconjunctival and mitomycin C intraoperatively was effective in preventing recurrence up to 14 months based on follow-up. Our different results from other studies possibly occurred because we could test changes in mRNA IL-1 expression in blood but not in conjunctival tissue because we did not have a baseline for pterygium tissues. Many factors could influence the systemic condition, and this was subject to biases.

In the comparison between groups, as shown in Table 2, there were significant differences between the bevacizumab and placebo groups (p < 0.05). Bevacizumab can inhibit VEGF as well as the proliferation of fibrovascular tissue. VEGF is a key factor in remodelling wound healing tissue.^24^ IL-1 is a proinflammatory mediator that influences the inflammatory process and promotes immune and growth responses. The growth of a new capillary as a response to angiogenesis plays an important role as a response to injury, such as in wound healing. These processes can be suppressed by a vascular growth factor.^25^, ^26^, ^27^, ^28^ In our study, we used a triamcinolone subconjunctival that can inhibit inflammation and bevacizumab, which inhibits angiogenesis, and found that the mRNA expression of IL-1 in the bevacizumab group was higher than that in the triamcinolone group. Therefore, bevacizumab seemed more effective than triamcinolone in preventing recurrence after excision. Accordingly, pre-surgery injection of 20 mg of triamcinolone and 2.5 mg of bevacizumab into the subconjunctiva was effective in suppressing inflammation in pterygium patients. Kang et al.^29^ found that the inhibitory effect of anti-VEGF in the treatment of corneal neovascularization was not significant, although triamcinolone and bevacizumab were combined.

The results reported here have some limitations. The weakness of this study was that only 15 subject eyes were studied. The participants were observed until only one month after surgery. Because pterygium recurrence was highly locally related, our results could not support the effect of both triamcinolone and bevacizumab in reducing the recurrence risk of pterygium after excision because we only observed recurrence for one month after surgery. We were unable compare pterygium tissue before surgery due to ethical factors, so we cannot compare it as we did in the blood sample, before and after giving an injection.

Therefore, this study requires further research on the role of mRNA IL-1 expression and what factors are involved in it.

## Conclusion

The expression of IL-1 mRNA in the bevacizumab group was higher than that in the triamcinolone and placebo groups, so Bevacizumab seems more effective than triamcinolone in preventing recurrence after excision.

## Recommendation

Further follow-ups are needed because in this study, we only followed up with patients twice (once after seven days and then one month after surgery) and we did not observe recurrence during this period. We suggest evaluating pterygium tissue after treatment.

## Source of funding

This research did not receive any specific grant from funding agencies in the public, commercial, or non-profit sectors.

## Conflict of interest

The authors have no conflicts of interest to declare.

## Ethical approval

This study was approved by the Institutional Review of Research Board Ethics Committee of Medical Faculty of Hasanuddin University, Makassar, South Sulawesi, Indonesia, No. 416/H4.8.4.5.31/PP36-KOMETIK/2018; Date: 21 June 2018.

## Patients consent

Written informed consent for injection, surgery, blood sampling, and pterygium sampling was obtained from all participants according to the principles outlined in the Declaration of Helsinki.

## Authors' contributions

PP, BB, MH, MNM, AMI, and RM conceived and designed the study, conducted the research, provided materials, collected and organized the data, drafted the manuscript, analyzed and interpreted the data. All authors critically reviewed and approved the final draft article and are responsible for the content and similarity index of the manuscript.
